# Mulberry Extract Attenuates Endothelial Dysfunction through the Regulation of Uncoupling Endothelial Nitric Oxide Synthase in High Fat Diet Rats

**DOI:** 10.3390/nu11050978

**Published:** 2019-04-28

**Authors:** Geum-Hwa Lee, The-Hiep Hoang, Eun-Soo Jung, Su-Jin Jung, Soo-Wan Chae, Han-Jung Chae

**Affiliations:** 1 Non-Clinical Evaluation Center, Biomedical Research Institute, Chonbuk National University Hospital, Jeonju 54907, Chonbuk, Korea; heloin@jbnu.ac.kr (G.-H.L.); drhiep.ydhue@gmail.com (T.-H.H.); 2 Clinical Trial Center for Functional Foods (CTCF2), Chonbuk National University Hospital, Jeonju 54907, Chonbuk, Korea; esjung@jbctc.org (E.-S.J.); sjjeong@jbctc.org (S.-J.J.); 3 Department of Pharmacology, Chonbuk National University Medical School, Jeonju 54896, Chonbuk, Korea; 4 Institute of New Drug Development, School of Medicine, Chonbuk National University, Jeonju 54907, Chonbuk, Korea

**Keywords:** mulberry, vascular function, high-fat diet, HUVECs

## Abstract

Dyslipidemia is associated with endothelial dysfunction, which is linked to nitric oxide (NO) biology. The coupling of endothelial NO synthase with cofactors is a major step for NO release. This study is aimed to investigate the vascular pharmacology effects of mulberry in rat thoracic aorta and human vascular endothelial cells. In vitro, we investigated the protective effects of the mulberry extract and its main component cyanidin-3-rutinoside (C-3-R), against oxidized low-density lipoprotein (ox-LDL)-induced endothelial nitric oxide synthase (eNOS) uncoupling. Whereas ox-LDL significantly decreased NO levels in endothelial cells, mulberry extract, and C-3-R significantly recovered NO levels and phospho-eNOS Thr495 and Ser1177 expression. In vivo, mulberry was administered to 60% of high-fat diet (w/w)-fed Sprague Dawley (SD) rats for six weeks, in which endothelium-dependent relaxations were significantly improved in organ bath studies and isometric tension recordings. Consistently, aortic expressions of phospho-eNOS and nitrotyrosine were increased. Mulberry also raised serum NO levels, increased phosphorylation of eNOS, and reduced nitrotyrosine and intracellular reactive oxygen species (ROS) in aortas, showing that mulberry preserves endothelium-dependent relaxation in aortas from high-fat diet rats. We suggest that this effect is mediated through enhanced NO bioavailability, in which the regulation of ROS and its reduced eNOS uncoupling are involved.

## 1. Introduction

Oxidized low-density lipoprotein (ox-LDL) contains various oxidized lipid molecules and is related with hyperlipidemia and atherosclerosis [1]. The same oxidized lipids found in ox-LDL are also formed in apoptotic cells and are present in circulation and tissues under pathological conditions. Excessive lipids, particularly ox-LDL, cause endothelial dysfunction and ultimately result in multiple cardiovascular diseases [2]. Under shear stress, normal endothelium produces vasodilators, including nitric oxide (NO), to control the blood pressure in blood vessels. In hyperlipidemia, this property is lost and is termed as endothelial dysfunction in ox-LDL-induced senescent models of endothelial cells [3,4]. Reactive oxygen species (ROS) have been also implicated in ox-LDL formation and endothelial dysfunction [5]. ox-LDL plays an important role in the early inflammatory process, accelerating endothelial dysfunction and inducing endothelial cell death, a process that can lead to atherosclerotic lesions [6]. An association between the impairment of endothelial nitric oxide synthase (eNOS) function and a decrease of NO production in non-alcoholic fatty liver disease (NAFLD) has been suggested [7,8]. In the endothelial contraction process, ROS contributes to the eNOS-uncoupled cardiovascular dysfunction where peroxynitrite (ONOO^−^) accumulates, amplifying the detrimental effects of ROS. Phenolic compounds, flavonoids, and anthocyanins, active substances in vegetables and fruits, have strong antioxidant effects and are now considered as a potential strategy for preventing endothelial dysfunction and cardiovascular pathological conditions [9,10]. Mulberry is rich in phenols, flavonoids, anthocyanins, and many other antioxidants, and has been applied to foods and pharmaceuticals because of its pharmacological effects. Thus, the main purpose of this study is to investigate the effects of mulberry supplementation on high-fat diet-induced adverse effects on the lipid profile, in vivo serum ox-LDL, and endothelial contraction. In this experiment, mulberry was given to high-fat diet-fed rats, as a model of experimental dyslipidemia and oxidative stress, especially against the endothelium. We report that the administration of mulberry prevents endothelial dysfunction in rats fed a high-fat diet, an animal model for lipid metabolism imbalance.

## 2. Materials and Methods 

### 2.1. Ethics Statement

All experimental animals used in this study were cared for under a protocol approved by the Institutional Animal Care and Use Committee of Chonbuk National University Hospital (cuh-IACUC-2016-26). All methods were performed in accordance with the guidelines and regulations set and approved by Chonbuk National University Hospital. 

### 2.2. Mulberry Extract

Mulberries were obtained from the South Buan Agricultural Cooperative Federation (Buan, Korea) in 2016. A voucher specimen (ID201803) has been deposited at the Herbarium of Department of Pharmacology, Chonbuk National University Medical School, Jeonju, Korea. Mulberry was extracted with 10 volumes of 70% ethanol at 50 °C for 3 h using a DH-M03 accelerated solvent extractor (D.M ENGINEERING Corporation, Siheung, Korea). The extracts were filtered using filter cartridges (1 µm). These filtrates were concentrated and spray dried. The content of cyanidin-3-rutinoside (C-3-R) in the extracts was calculated from the relevant peak area using an external standard method and quantified as 0.15%.

### 2.3. High-Performance Liquid Chromatography Analysis of Mulberry Extract

The components of the mulberry extract were analyzed using an Agilent 1260 Infinity high-performance liquid chromatography (HPLC) system (Agilent, Santa Clara, CA, USA) with an XDB (eXtra Densely Bonded) C18 column (4.6 × 250 mm, 5 µm; Agilent, Santa Clara, CA, USA). The mobile phase was composed of 10% formic acid in Distilled Water (DW) (A) and acetonitrile:methanol: DW: Formic acid = 22.5:22.5:45:10 (B). The gradient program (A:B) was as follows: 0 min, 85:15; 25.45 min, 64:36; 27 min, 85:15; and 30 min, 85:15. The flow rate was 1 mL/min, the injection volume was 10 µL, and the column temperature was maintained at 35 °C. The detector was set at the wavelength of 530 nm. The standard (C-3-R) for HPLC analysis was obtained from Sigma-Aldrich (St Louis, MO, USA). The C-3-R content was determined with a validation method using dilutions of each standard at concentrations ranging from 5.25 ppm to 238.00 ppm injected into the HPLC system (correlation coefficient 0.999). In the mulberry-associated endothelial function study, analysis of the main constituents of the mulberry extract was performed at first. In HPLC analysis, we quantified the contents of components from mulberry extracts (0.15%). C-3-R was identified as the main components from the mulberry extracts.

### 2.4. Animal Grouping and Experimental Protocol

Male Sprague-Dawley rats that were 7-weeks-old and weighing 250–270 g were obtained from Orient Science Co (Seongnam, Korea). Rats were maintained in a 12:12 h light:dark cycle (lights on at 06:00) in a stainless steel wire bottom cage and acclimatized under laboratory conditions for at least one week before the experiments. The normal chow diet (NCD) group was fed a standard diet, whereas the high-fat diet (HFD) group was fed a calorie-rich diet of 1% cholesterol, 18% lipid (lard), 40% sucrose, 1% AIN-93G vitamins, and 19% casein, with the same fiber and minerals as the control group’s diet. Rats in the mulberry groups were fed 100 mg/kg with the HFD.

Group 1 (*n* = 10 each): NCD-group, rats received double-distilled water (DDW)
Group 2 (*n* = 10 each): NCD plus mulberry-group, rats received DDW with mulberry (100 mg/kg)
Group 3 (*n* = 10 each): 60% HFD-group, rats received DDW
Group 4 (*n* = 10 each): 60% HFD plus mulberry-group, rats received DDW with mulberry (100 mg/kg)

Experiments were terminated after 10 weeks. Rats were anesthetized with ketamine (Yuhan Yanghyang company, Seoul, Korea) and cervical dislocation was performed. 

### 2.5. Biochemical Analysis 

Plasma levels of total cholesterol (TC) and triglyceride (TG) were measured using a quantification kit (Asan Pharmaceutical, Seoul, South Korea) following the instruction manual. TC and TG levels were measured spectrophotometrically, and absorbance was read with an Enzyme-linked immunosorbent assay (ELISA) Reader at 500 and 510 nm. Oxidized LDL (Life Span Biosciences, Seattle, DC, USA) and LDL-cholesterol (Biovision, Milpitas, CA, USA) were measured using commercially available kits. Oxidized LDL and LDL-cholesterol levels were measured spectrophotometrically and absorbance was read with an ELISA reader at 450 and 570 nm.

### 2.6. Human Umbilical Vein Endothelial Cell Culture

Human umbilical vein endothelial cells (HUVECs) were purchased from the American Type Culture Collection (Manassas, VA, USA) and cultured in endothelial basal medium 2, supplemented with endothelial growth cell medium 2 (EGM-2) (Lonza, Basel, Switzerland) under 5% CO_2_ at 37 °C. When HUVECs had grown to 85–90% confluence in 6-well plates, they were incubated with 20 μg/mL of ox-LDL with or without mulberry extract (500 μg/mL), C-3-R (50 μg/mL) for 24 h. In experiments, HUVEC cells were used from passages three to nine.

### 2.7. Immunoblotting 

Aorta ring homogenates and cell lysates were separated by 7% sodium dodecyl sulfate polyacrylamide gel electrophoresis (SDS-PAGE) and transferred to Polyvinylidene difluoride (PVDF) membranes. After blocking with 5% skim milk, the blot was probed with primary antibodies against p-eNOS, eNOS (Cell Signaling Technologies, Inc., Danvers, MA, USA), nitrotyrosine (Abcam, Inc., Cambridge, MA, USA) and β-actin (Santa Cruz Biotechnologies, Inc., Santa Cruz, CA, USA). Anti-rabbit IgG Horseradish peroxidase (HRP) (Invitrogen) or anti-mouse IgG HRP (Invitrogen) diluted 1:5000, were used as secondary antibodies (Abs), and the immunoblots were visualized with the Enhanced chemiluminescence (ECL) system (Bio-Rad, Hercules, CA, USA).

### 2.8. Lucigenin-Enhanced Chemiluminescence Assay 

Lucigenin is an acridinium compound that releases light when interacting with O_2_^−^. We used lucigenin for O_2_^−^ production analysis as previously described [11]. We used lucigenin for O_2_^−^ production analysis as described previously. Briefly, after removing cell debris, a 50 μL aliquot of the cell homogenate was added to a Krebs-4-(2-hydroxyethyl)-1-piperazineethanesulfonic acid HEPES) buffer (99 mM NaCl, 4.7 mM KCl, 1.2 mM MgSO_4_, 1 mM KH_2_PO_4_, 1.9 mM CaCl_2_, 25 mM NaHCO_3_, 11 mM glucose, 20 mM HEPES, pH 7.4). Luminescence was detected using Spectra Max Microplate Luminance (Molecular Devices, Sunnyvale, CA, USA). The final value is expressed in relative light units normalized to protein concentration. The average chemiluminescence observed for 10 min was used to evaluate the production of O_2_^−^

### 2.9. Chemiluminescent Detection of NO Production

Accumulated nitrite in the cell culture media was used as an indicator of NO production and determined by the Griess reaction, as described previously [12]. Supernatants (100 μL/well) were then collected, mixed with equal volumes of nitrate/nitrite assay kit (Sigma Chemical), and incubated at room temperature for 10 min. Nitrite concentration was determined by absorbance at 540 nm. The absorbance of the sample was measured at 540 nm and compared with the standard curve.

### 2.10. NO Imaging of Living Endothelial Cells 

To quantify the steady-state level of NO in live HUVECs, a NO-sensitive phosphor dye, diamino-fluorescein-2-diacetate (DAF-2DA, Invitrogen/Molecular Probes, Eugene, Oregon, USA), was applied. Cells were loaded with DAF-DA for 30 min at 37 °C. Cellular areas were quantified by DAF-DA staining with background fluorescence using epifluorescence (Applied Precision Delta Vision Elite, Applied Precision Inc., Issaquah, WA, USA).

### 2.11. Cellular Production of ROS Using Dihydroethidium (DHE) and MitoSox 

The level of intracellular ROS including O_2_^−^ was detected using an oxidation-sensitive fluorescent probe dye, dihydroethidium (DHE, Ex/Em = 518/605 nm, Invitrogen/Molecular Probes, Eugene, Oregon, USA). Cells and aortic rings were washed with PBS, incubated with 20 μM DHE for 30 min at 37 °C, and then added to 5 μM of MitoSox for 30 min. For quantitative measurement, the cells were scanned with a 20× objective. Cellular areas were quantified by DHE and MitoSox staining using background fluorescence and epifluorescence (Applied Precision Delta Vision Elite, Applied Precision Inc., Issaquah, WA, USA).

### 2.12. DHR Oxidation

DHR oxidation was used to evaluate peroxynitrite production of cells exposed, as previously described [13]. Cells were grown in a confocal dish, and liver paraffin tissue was replaced with 0.5 mL of Dulbecco’s phosphate-buffered solution (dPBS) containing 20 μM DHR (Invitrogen, Carlsbad, CA, USA) at 37 °C for 30 min. The detection of rhodamine 123 (ex 485 nm/em 520 nm), an oxidation product of DHR, after exposure to the different experimental conditions was followed online in epifluorescence (Applied Precision Delta Vision Elite, Applied Precision Inc., Issaquah, WA, USA) with the subtraction of background fluorescence.

### 2.13. Lipid Peroxidation

To measure lipid peroxidation, the concentrations of the lipid peroxidation products, malondialdehyde (MDA) and 4-hydroxynonenal (4-HNE), were measured using the BIOXYTECH LPO-586 commercial kit (Oxis International Inc., Portland, OR, USA) according to the manufacturer’s protocol.

### 2.14. Analysis of Vascular Function

The aortic rings were rapidly dissected and placed into a Krebs buffer. The aortic rings were carefully removed and the separated descending aorta was cut into rings (3–4 mm in length). The aorta were suspended in 95% O_2_/5% CO_2_ aerated organ chambers filled with modified Krebs buffer (118 mM NaCl, 1.2 mM MgCl_2_, 11.2 mM NaH_2_PO_4_, 4.7 mM KCl, 1.2 mM Na_2_SO_4_, 25 mM NaHCO_3_, 10 mM glucose; pH 7.4) [14]. Changes in isometric tension were recorded using a force displacement transducer (Grass FT 0.3, Quincy, MA, USA) connected to a Power Lab system 400 (ML 118, PowerLab, AD Instruments, Medford, MA, USA) and stored in a computer. Before contraction, a tension of 2.0 g was given to the aorta ring for 30 min. During this period, the Krebs-Ringer bicarbonate solution was changed every 15 min. After the equilibration, aortic rings were challenged with 80 mM KCl. After washing and another 30 min equilibration period, contractile response was elicited by 1 μM phenylephrine. At the plateau of contraction, accumulative acetylcholine (ACh, 10^−9^–10^−4^ M) or sodium nitroprusside (SNP) was added into the organ bath to induce endothelium-dependent or -independent relaxation.

### 2.15. Immunohistochemical Analysis of eNOS Expression

The eNOS expression in the aorta were detected by immunohistochemistry. In immunolocalization of phospho-eNOS (Thr495) data, the p-eNOS was expressed specifically on the luminal side. 

### 2.16. Statistical Analysis 

Data were expressed as the mean ± standard error of the mean (SEM). The groups were compared using one-way ANOVA with Tukey post hoc comparison. *p* < 0.05 was considered statistically significant.

## 3. Results

### 3.1. Analysis of Compounds in Mulberry Extract 

**Figure 1:**
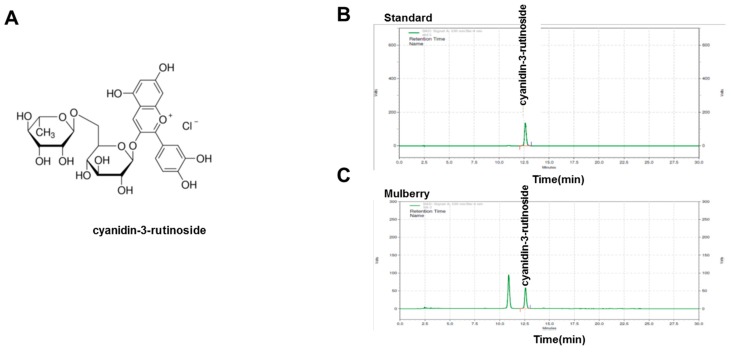
HPLC analysis of mulberry extracts. (**A**) Chemical structure of C-3-R. Chromatograms for C-3-R was analyzed for standard solution (**B**) and mulberry extracts (**C**). C-3-R, cyanidin-3-rutinoside.

In the mulberry-associated endothelial function study, analysis of the main constituents of mulberry extract was first performed. In HPLC analysis, we quantified the contents of components from mulberry extracts (0.15%). C-3-R was the main component of the mulberry extracts. In this study, we selected C-3-R as an effective component as well as a standardization component for the extraction procedure. Figure 1A shows the structure of C-3-R. Chromatogram data on the mulberry extracts with the C-3-R standard are also presented in Figure 1B,C. 

### 3.2. Mulberry Regulates HFD-Induced Lipid Dysmetabolism

**Figure 2:**
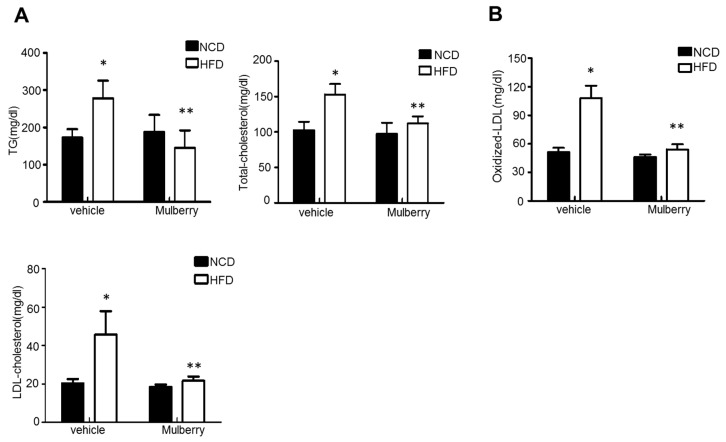
Mulberry prevents high-fat diet (HFD)-induced hepatic steatosis. Rats were fed a normal diet or a high-fat diet with or without 100 mg/kg mulberry for six weeks, and serum and liver were harvested. (**A**) Biochemical analysis of serum samples. Levels of triglycerides (TG), total cholesterol (TC) and low-density lipoprotein (LDL)-c were measured in the serum of rat in different experimental groups. Values are presented as mean ± SEM. (*n* = 6, * *p* < 0.05 versus the normal chow diet (NCD) group, ** *p* < 0.05 versus the HFD group) (**B**) Levels of oxidized-LDL were measured in the serum of rat in different experimental groups. Values are presented as mean ± SEM. (*n* = 6, * *p* < 0.05 versus the NCD group, ** *p* < 0.05 vs. the HFD group).

Several studies have reported the impairment of eNOS function and decrease of NO production in the progression of lipid dysmetabolic state [15,16,17]. As an endothelial dysfunction model to reveal the regulatory role of mulberry to the fatty liver, we established an animal model of fatty liver in which SD rats were fed with a 60% high-fat diet (HFD). As shown in Figure 2A, triglyceride (TG), total cholesterol (TC), and LDL-c (LDL-cholesterol) levels in serum were significantly increased in the HFD-group compared to the control group. After six weeks of treatment, serum levels of TG, TC, and LDL were significantly reduced in both the HFD plus mulberry-group compared to the HFD-group. The production of oxidized LDL (ox-LDL), an atherosclerosis-linked ROS generator, has also been confirmed. Expectedly, the ox-LDL was highly increased in the HFD-group, and was significantly regulated in the HFD plus mulberry-group (Figure 2B).

### 3.3. Effects of Mulberry on Oxidative Stress in Hyper-Lipidemic Rats

**Figure 3:**
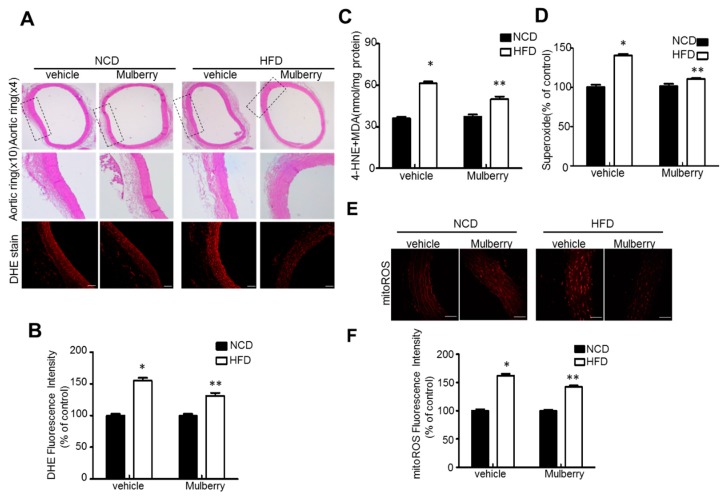
Mulberry inhibits oxidative stress in hyperlipidemic rats. Rats were fed a normal diet or a high-fat diet with or without 100 mg/kg mulberry for six weeks, and serum and aorta rings were harvested. (**A**) Aorta rings tissues retrieved six weeks after initial mulberry administration were subjected to hematoxyline-eosine (H&E) staining and DHE staining. Scale bars, 50 μm. (**B**) Quantification of fluorescence intensity for superoxide levels in aortas. Values are presented as mean ± SEM (*n* = 6, * *p* < 0.05 versus NCD-group; ** *p* < 0.05 versus HFD-group). (**C**) MDA plus 4-hydroxynonenal (4HNE) levels in aortic tissues were assessed, respectively. (**D**) Endothelial O_2_^−^ production was assessed as described in the Materials and Methods. (**E**) Representative images of MitoSox staining for mitochondrial superoxide anion production. (**F**) Quantification of fluorescence intensity for mitochondria ROS levels in aortas. Values are presented as mean ± SEM (*n* = 6, * *p* < 0.05 versus NCD-group; ** *p* < 0.05 versus HFD-group). DHE, dihydroethidium; NCD, normal control diet; HFD, high fat diet; MDA, malondialdehyde; ROS, reactive oxygen species.

We examined the effect of orally administered mulberry on oxidative stress in rats fed a high-fat diet. Superoxide was measured by the evaluation of superoxide indicator dihydroethidium (DHE). Expectedly, mulberry controlled the highly stimulated fluorescence in the aorta rings from the high fat diet-feeding condition (Figure 3A,B). Similarly, mulberry decreased lipid peroxidation and superoxide production in the aorta (Figure 3C,D). Indeed, an increase in the staining fluorescence signal in the aortic rings with MitoSox, an indicator of mitochondrial ROS production, indicates superoxide formation. (Figure 3E,F). MitoSox levels in the aorta rings were downregulated in HFD plus mulberry, compared to the HFD-groups, although there was no obvious change of vascular endothelial structure in the aortic tissues by the representative morphology analysis, i.e., hematoxyline-eosine (H&E) staining.

### 3.4. Effects of Mulberry on Vascular Endothelial Function in Hyperlipidemic Rats

**Figure 4:**
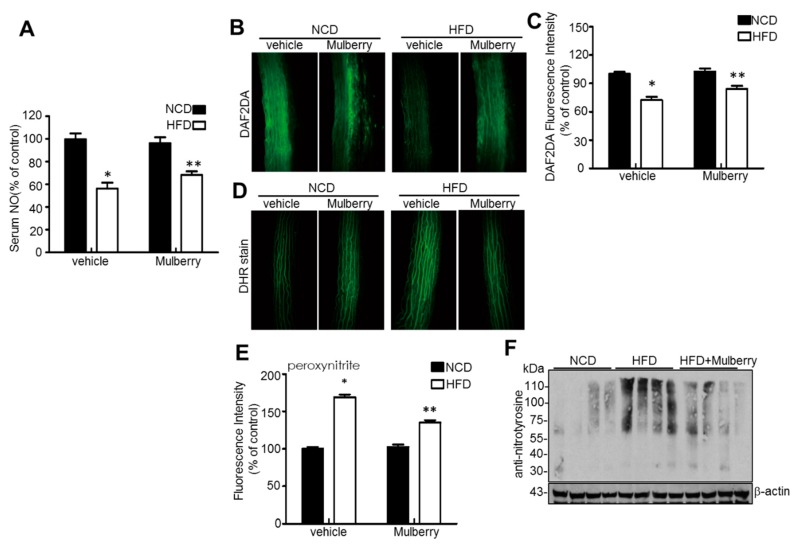
Mulberry inhibits oxidative stress in hyperlipidemic rats. Rats were given a normal diet or a high-fat diet with or without 100 mg/kg mulberry for six weeks, and serum and aorta rings were harvested. (**A**) Level of NO was measured in the serum of rat in different experimental groups. Relative quantification of NO in serum. Values are presented as mean ± SEM. (*n* = 6, * *p* < 0.05 versus the NCD group; ** *p* < 0.05 versus the HFD group). (**B**) NO levels in aortic tissues were assessed, respectively. Scale bars, 50 μm. (**C**) Quantification of DAF2DA fluorescence intensity for NO levels in aortas. Scale bars, 50 μm (*n* = 5, * *p* < 0.05 versus the NCD group; ** *p* < 0.05 versus the HFD group). (**D**) Representative images of DHR staining for peroxynitrite in aortas. (**E**) Quantification of DHR fluorescence intensity for peroxynitrite levels in aortas. Values are presented as mean ± SEM. (*n* = 6, * *p* < 0.05 versus the NCD group; ** *p* < 0.05 versus the HFD group). Nitrotryosine (**F**) and expression of β-actin in the aortas were determined by Western blotting. NO, nitric oxide; DAF2DA, diaminofluorescein-2 diacetate; NCD, normal control diet; HFD, high-fat diet; DHR, dihydrorhodamine.

Abnormalities in NO production by the vascular endothelium result in endothelial dysfunction, which occurs in atherosclerosis [18,19]. Thus, we measured NO in serum and isolated rat aortic rings and in the aortic rings by staining diamino-fluorescein-2-diacetate (DAF-2DA). In Figure 4A–C, the amounts of NO in serum and the aorta ring were significantly maintained in the HFD plus mulberry-group, compared to the HFD-group. The fluorescent probe dihydroxy-rhodamine (DHR) reacts with peroxynitrite-derived free radicals but not with O_2_^−^ or NO directly [20]. In addition, mulberry markedly attenuated peroxynitrite (Figure 4D,E) and reduced levels of nitrotyrosine (Figure 4F) in aortas. This data suggests that dyslipidemia-related abnormal superoxide anion accumulation reduces NO bioavailability and converts NO into peroxynitrite, which causes endothelial dysfunction [21], whereas the mulberry controls the dyslipidemia-related NO disturbance. 

### 3.5. Mulberry Recouples eNOS in Rats In Vivo

**Figure 5:**
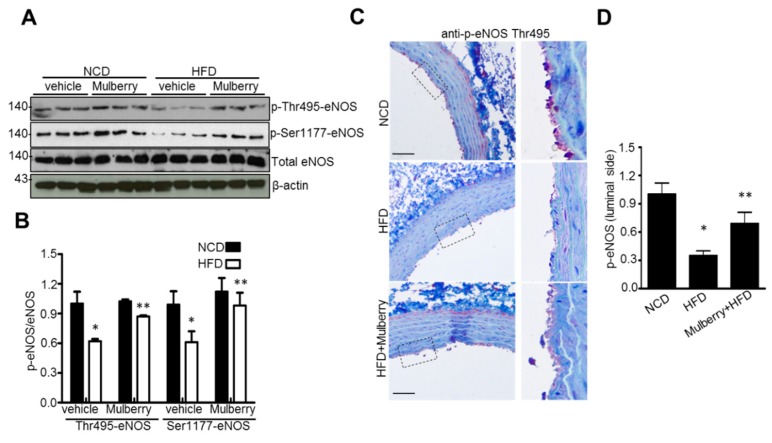
Mulberry recouples eNOS in hyperlipidemic rats. Rats were given a normal diet or a high-fat diet with or without 100 mg/kg mulberry for six weeks, and aorta rings were harvested. Phosphorylation of Ser1177 and Thr495 (**A**) and expression of total eNOS and β-actin in the aorta (**B**) were determined by Western blotting. Values are presented as mean ± SEM. (*n* = 3; * *p* < 0.05 versus the NCD group; ** *p* < 0.05 vs. the HFD group) (**C**) Representative images of the immunohistochemical staining of phosphorylation of eNOS at Thr495 (p-eNOS) on aortic sections. Scale bars, 50 μm. (**D**) Quantification of phosphorylation of eNOS at Thr-positive luminal side. Values are presented as mean ± SEM. (*n* = 6. * *p* < 0.05 versus the NCD group; ** *p* < 0.05 versus the HFD group). NCD, normal control diet; HFD, high-fat diet.

To illustrate whether eNOS recoupling is involved in the anti-hyperlipidemic effects of mulberry in vivo, we examined the function of eNOS in hyperlipidemic rats. The mechanisms involved in eNOS coupling include phosphorylation of eNOS on Thr-495 and Ser-1177 [22,23]. Mulberry supplement at the aortic rings increased the phosphorylation of eNOS on Thr495 and Ser-1177, compared to the HFD-group (Figure 5A,B). Consistently, Mulberry recovered eNOS on Thr-495 phosphorylation in the aortic luminal side of high-fat diet rats (Figure 5C,D), showing the role of Mulberry on the eNOS coupling through the regulation of eNOS phosphorylation in hyperlipidemia. 

### 3.6. Mulberry Prevents Endothelial Dysfunction in Aortic Arteries Isolated from Rats Ex Vivo 

**Figure 6:**
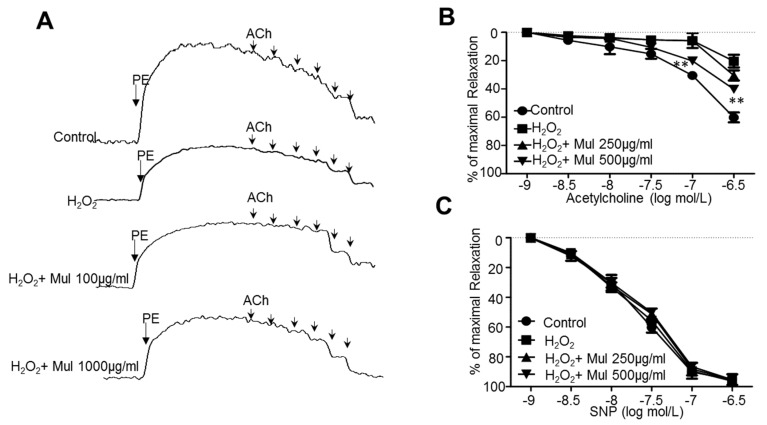
Mulberry prevents endothelial dysfunction in aortic arteries isolated from rats ex vivo. Aortas were exposed to 0.2 mmol/L H_2_O_2_ for 2 h in which water or mulberry (250 or 500 μg/mL) was treated during the subsequent 30 min in aortas. (**A**,**B**) The endothelium-dependent relaxation induced by acetylcholine (ACh) was analyzed and (**C**) endothelium-independent relaxation was also confirmed by sodium nitroprusside (SNP) in an organ chamber. All data are expressed as mean ± SEM. (*n* = 5; ** *p* < 0.05 versus H_2_O_2_).

**Figure 7:**
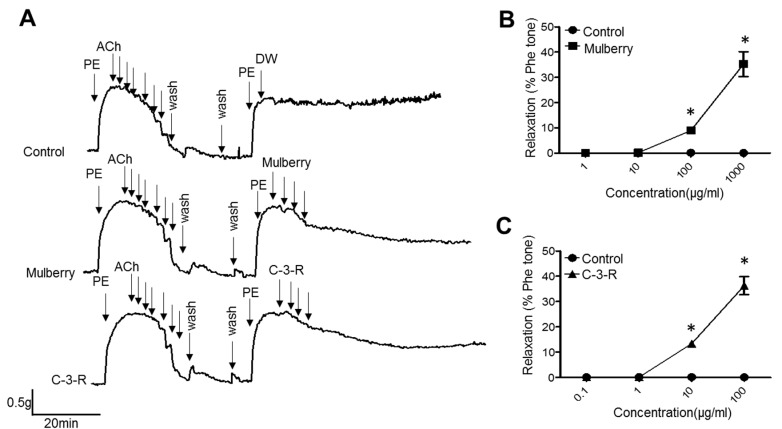
Extract and C-3-R enhances endothelial relaxation in aorta rings. (**A**) Representative tracings show that relaxation of rat thoracic aorta is induced by cumulative addition of 1 to 1000 μg/mL mulberry and 0.1 to 100 μg/mL C-3-G and C-3-R at the plateau of the phenylephrine (1μM PE)-induced contraction, followed by application of acetylcholine (ACh, 10^−11^ to 10^−6^ M). Concentration-response curve of endothelium-intact aortic ring relaxation following mulberry treatment (**B**) and C-3-R (**C**) was expressed as a % of the PE-induced contraction. Results are expressed as percent relaxation ± SEM. (*n* = 5; * *p* < 0.05 vs. control). PE; phenylephrine

C-3-R is one of the major components of mulberry, composing the mulberry components of approximately 0.15% here. Since the structure of C-3-R includes several hydroxyl residues, the bioavailability seems not to be high and stable [24]. In addition, the effect of mulberry has been suggested to be originated from the unidentified synergistic effect of each glucoside such as C-3-R or C-3-G [25]. Therefore, it was not easy to determine the concentration of C-3-R, based upon the concentration of mulberry. Similarly to previous reports studying the herbal extracts and their components [26], we treated the mulberry and C-3-R with the ratio of “1:0.1”. Especially in Figure 7, the polarity of the C-3-R structure might be a critical factor in the bath-based ex vivo experiment. Therefore, we applied the ratio of concentration between mulberry and C-3-R “1:0.1”. We then tested whether mulberry protects vascular endothelial functions by incubating rat aortic rings with H_2_O_2_ ex vivo. The endothelium-intact thoracic aorta rings were obtained from seven-week-old male rats. As shown in Figure 6A,B, exposure of the aortic rings to H_2_O_2_ dramatically impaired acetylcholine (ACh)-induced endothelium-dependent relaxation, which was concentration-dependently reversed by mulberry. However, endothelium-independent relaxations to SNP were similar among the four groups (Figure 6C). 

In the endothelium-intact thoracic aorta rings in seven-week-old male rats, mulberry extract and C-3-R caused a potent dose-dependent vaso-relaxation effect on the 1.0 μM phenylephrine PE)-contracted aorta rings. The representative aortic ring contraction/relaxation graph was shown (Figure 7A). Mulberry relaxed rat arteries were clearly concentration-dependently. A rapid and almost complete relaxation reached at the concentration of 1000 μg/mL. When the contraction by PE (10^−5^ mol/L) was stabilized, C-3-R and mulberry extracts were added. In the endothelium-intact aortic ring preparations, mulberry (1 to 1000 μg/mL) relaxed isolated rat aorta in a concentration-dependent manner with the maximum value of 40 ± 8% at a concentration of 1000 μg/mL (Figure 7B). C-3-R (0.1 to 100 μg/mL) relaxed isolated rat aorta in a concentration-dependent manner with the maximum value of 39 ± 6% at a concentration of 100 μg/mL (Figure 7C). In the endothelium-intact aorta, mulberry and C-3-R enhanced vascular tension in a concentration-dependent manner indicating endothelium- dependent relaxation also occurs.

### 3.7. The Main Component of Mulberry Extract and C-3-R, Regulates Endothelial Function in HUVECs

**Figure 8:**
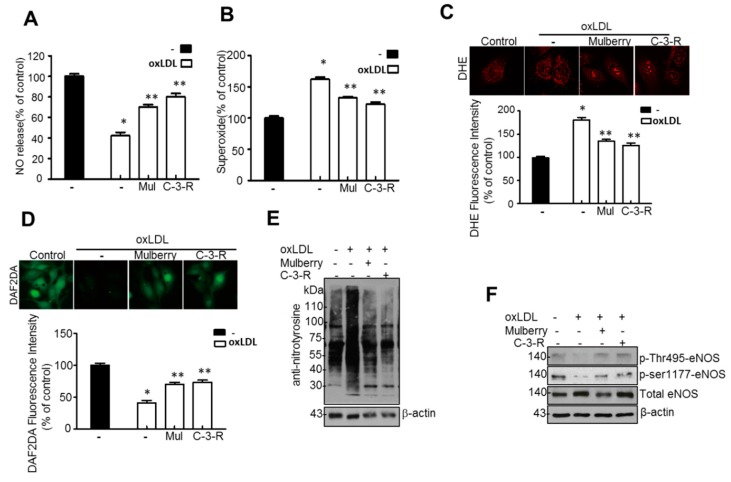
Mulberry improves endothelial cell functions in ox-LDL-treated human umbilical vein endothelial cells (HUVECs). HUVECs were treated with 50 μg/mL C-3-R and 500 μg/mL mulberry, then incubated with 20 μg/mL ox-LDL for another 24 h. NO production (**A**) and endothelial O_2_^−^ production (**B**) were assessed as described in the materials and methods. Values are presented as mean ± SEM. (*n* = 3; * *p* < 0.05 versus control; ** *p* < 0.05 vs. ox-LDL) (**C**) Representative images of DHE staining for O_2_^−^ production. Values are presented as mean ± SEM. (*n* = 3; * *p* < 0.05 versus control; ** *p* < 0.05 vs. ox-LDL) (**D**) Quantification of DHR fluorescence intensity for peroxynitrite levels. Values are presented as mean ± SEM. (*n* = 3; * *p* < 0.05 versus control; ** *p* < 0.05 vs. ox-LDL). Nitrotryosine (**E**) and expression of β-actin in HUVECs were determined by Western blotting. Phosphorylation of eNOS at Thr495 and Ser1177 (**F**) and expression of total eNOS and β-actin in the HUVECs were determined by Western blotting. HUVEC, human umbilical vein endothelial cells; C-3-R, cyanidin-3-rutinoside; ox-LDL, oxidized low-density lipoprotein; NO, nitric oxide; DHE, dihydroethidium; DHR, dihydrorhodamine.

NO release and O_2_^−^ production were analyzed in ox-LDL-treated HUVECs treated with 50 μg/mL C-3-R. Ox-LDL decreased NO level in HUVECs, while the addition of C-3-R abolished this effect (Figure 8A). C-3-R also significantly blocked ox-LDL-induced O_2_^−^ production (Figure 8B), suggesting that they effectively inhibit O_2_^−^ production, thereby controlling the release of NO. DHE staining, which is extensively used to evaluate ROS production, verified that mulberry significantly attenuated cellular ROS production in aortas (Figure 8C). In addition, mulberry markedly attenuated peroxynitrite (Figure 8D) and reduced levels of nitrotyrosine (Figure 8E) in HUVECs. We next examined eNOS phosphorylation status in the presence of C-3-R. Ox-LDL significantly reduced the phosphorylation of eNOS Thr495 and Ser-1177, while C-3-R treatment of HUVECs increased eNOS Thr495 and Ser1177 phosphorylation (Figure 8F). Consistently, the isolated main component of the mulberry extracts, C-3-R, regulated eNOS uncoupling by restoring eNOS phosphorylation in ox-LDL-treated human endothelial cells.

## 4. Discussion

In this study, through six weeks of uncontrolled hyperlipidemia, rats showed evidence of vascular endothelial dysfunction such as impaired NO bioactivity and its associated eNOS uncoupling status, whereas mulberry regulated the dysfunctional stress. This study also revealed that mulberry extract inhibits the eNOS uncoupling-associated endothelial dysfunction both in vivo and in vitro, providing support for the potential usefulness of mulberry and its main components in the hyperlipidemia-related vascular endothelial dysfunction. Concentrations of phenolic components and flavonoids are high in mulberry extract. In this study, mulberry extract exhibited a high concentration of C-3-R (Figure 1). The two major anthocyanins present in the *Rubus* fruits produced in Korea are C-3-G and C-3-R [27]. These highly contained anthocyanins in mulberry are one of the subgroups of flavonoids, which have various physiologically active functions [28]. Through an anti-oxidative effect, the anthocyanins were shown to dramatically ameliorate the formation of ox-LDL in HUVEC cells [11]. C-3-R showed a consistent effect of regulation against the disturbance of NO release and eNOS p-Thr and p-Ser phosphorylation (Figure 8), indicating the components contribute to the maintenance of cardiovascular endothelium against ox-LDL-associated stresses. As a traditional herbal medicine, mulberry has been used in the prevention and the treatment of cardiovascular diseases [29]. Recent studies have shown that mulberry extracts and its active compounds, including anthocyanin, have biochemical properties such as anti-hyperlipidemic, anti-atherosclerotic, antioxidant, anti-diabetic, and immunomodulatory effects [30,31,32,33]. Anthocyanins in many berries including wild blueberries, wild bilberry, cranberries, elderberries, raspberry seeds, and strawberries, which significantly improve the incidence of atherosclerosis by reducing the formations of oxidized-LDL and foam cells. It was also reported that higher intakes of anthocyanins including C-3-R and C-3-G are associated with a reduced risk of hypertension and chronic hyperglycemia-induced endothelial dysfunction, controlling systemic inflammation and cardiovascular diseases [34,35,36]. In these studies, the antioxidative characteristics of the berry extract and its components have been focused on as one of the main mechanisms. These abundant anthocyanins seem to contribute to the recovery of endothelial function through their antioxidative function, finally linked to the maintained nitric oxide bioactivity. The key finding from this study is that the effect of mulberry against endothelial dysfunction is related to the recovery of NO and eNOS activity in dyslipidemia conditions. We found that mulberry recovers the disrupted endothelial NO generation and eNOS activity (as determined by changes in phosphorylation status) under hyperlipidemia. The anthocyanin-abundant mulberry edited the dyslipidemia parameters TG, cholesterol, and LDL, and also regulated the endothelial oxidative lipid criteria ox-LDL, an oxidized status of LDL in the hyperlipidemic condition (Figure 2). Expectedly, the oxidative levels in aortas were downregulated in the high-fat diet plus mulberry-feeding condition compared to the HFD-group, although there was no obvious change of vascular endothelial structure in the aortic tissues of each group as indicated by H&E staining (Figure 3A). Aortic rings of HFD rats treated with mulberry resulted in the inhibition of endothelial mitochondrial superoxide anion production and intracellular ROS (Figure 3B–D), indicating that oxidative stress is amplifying into main subcellular organelles with severe oxidative stress. Considering that oxidative stress induces endothelial dysfunction primarily due to rapid oxidative inactivation of NO by excess superoxide [37], peroxynitrite formation may be another enhancing signaling messenger toward the disruption of NO bioavailability, another hypothesis in this study. Expectedly, the regulation of ONOO^−^ was also observed in the mulberry-treated group under the HFD condition compared to the non-treated group under the HFD condition (Figure 4). eNOS coupling, a post-translational complex formation-linked NO production step, was also hampered by the active electron transformation radicals including superoxide anion and the associated peroxynitrite in the HFD condition, whereas mulberry reversed this disruption (Figure 5A,B), suggesting that the mechanism of protection against eNOS uncoupling is likely due to the antioxidant properties of mulberry extracts. This is most likely facilitated by the ability of mulberry extract to prevent ox-LDL-induced reductions in phospho-eNOS Thr495 and Ser1177, thereby inhibiting ox-LDL-induced eNOS uncoupling. Among the studied phosphorylation of eNOS sites, Thr495 and Serine 114, 615 and 633, the phosphorylation of Ser1177 or Thr495 is particularly important [38]. Although there has been debate about the role of Thr495 phosphorylation about NOS activity [22,23], the role of Ser1177 phosphorylation has been relatively well established as a positive post-translational modification for NOS activity [39]. Here, in the presence of mulberry, the phosphorylation of Thr495 and Ser1177 rather increased, enhancing NO release, consistent to a recent study of mulberry [11]. The eNOS uncoupling has been implicated in a number of vascular diseases, such as hypertension [3], atherosclerosis [15,40], and diabetes [30]. When persistent oxidative stress renders uncoupled eNOS, such that it no longer produces NO but superoxide [41,42], mulberry is also expected to control eNOS uncoupling, leading to superoxide anion capture and maintaining NO and eNOS coupling. In this study, we found that the endothelial action of mulberry is mediated by eNOS recoupling, suggesting a therapeutic and preventive strategy against many molecular phenomena that are the source of endothelial damage [37,43]. eNOS uncoupling was reported to be significantly upregulated in hyperlipidemia mice and endothelium-dependent relaxation was markedly suppressed [16]. Our study also shows that the reduction in eNOS uncoupling is associated with the enhancement of acetylcholine-induced endothelium-dependent aortic relaxation found in HFD plus mulberry rats (Figure 6 and Figure 7). The results suggest that the increased endothelium-dependent aortic relaxation in the mulberry could be related to the increased vascular reactivity beyond its vasoconstriction property. 

## 5. Conclusion

In summary, our study indicates that mulberry improves endothelial function and eNOS coupling after high-fat diet conditions. We propose that mulberry polyphenols, such as anthocyanins, are at least partly responsible for these positive effects by reducing oxidative stress and restoring the eNOS coupling/NO pathway. These results could lead to the development of novel strategies to prevent complications related to vascular diseases.

## Abbreviations

ox-LDL	Oxidized low-density lipoprotein
eNOS	endothelial nitric oxide synthase
C-3-G	cyanidin-3-O-β-d-glucopyranoside
C-3-R	cyanidin-3-rutinoside
NAFLD	non-alcoholic fatty liver disease
HUVECs	human umbilical vein endothelial cells
NO	nitric oxide
ONOO^−^	peroxynitrite
ROS	reactive oxygen species
NCD	normal control diet
HFD	high-fat diet
TG	triglyceride
DAF-2DA	diamino-fluorescein-2-diacetate
DHE	dihydroethidium
PE	phenylephrine
ACh	acetylcholine
SNP	sodium nitroprusside
MDA	malondialdehyde
DHR	dihydrorhodamine.
